# New soliton dynamics revealed in the normal dispersion region

**DOI:** 10.1038/s41377-022-00748-1

**Published:** 2022-03-23

**Authors:** Kebin Shi

**Affiliations:** State Key Laboratory for Mesoscopic Physics and Frontiers Science Center for Nano-optoelectronics, School of Physics, Peking University, Beijing, 100871 China

**Keywords:** Ultrafast photonics, Solitons

## Abstract

Birefringence-involved phase matching is demonstrated to be a novel mechanism to generate transform limited solitary pulses in an ultrafast mode-locking fiber laser cavity with normal dispersion.

Ultrafast mode-locking fiber lasers are becoming attractive light sources for a variety of applications including scientific research and industrial productions due to their unique advantages such as portability, alignment-robustness, better thermal management and power-scalability. With the fast-pacing scientific explorations and industrial progressions, there have been increasing demands on fiber lasers for higher pulse energy, shorter pulse duration, better power-scalable mode-locking mechanisms and more controllable intra-pulse phase dynamics. Recently the developments of ultrafast fiber lasers have gained rapid advances by precisely tailoring the intracavity spectral and temporal properties for mode-locking oscillators and subsequent amplifiers, which essentially involve the interplay between multiple physical characteristics as exemplified by nonlinear optical effects and dispersion^1^. With the interaction between nonlinear and dispersive effects, soliton dynamics^2,3^ has become one of the most predominant mechanisms for understanding complex ultrafast pulse evolutions and developing novel femtosecond fiber lasers.

Analogous to spatial solitary wave, temporal soliton formation in fiber laser cavity undergoes balance between nonlinear (self-phase modulation) and dispersive phase accumulations. In order to compensate positive phase accumulation induced by nonlinear propagation, conventional soliton needs to work in the wavelength range where the anomalous group velocity dispersion of standard silica fiber is accessible^4^. Moreover, restraining soliton pulse energy within tenth of nano-Joule to avoid solitary wave breaking poses further limitation on practical use of conventional soliton fiber lasers. To tolerate higher pulse energy and sequential larger nonlinear phase shift without solitary wave breaking, many efforts have been carried to design dispersion maps with alternate normal and anomalous fiber segments, which can support dispersion-managed solitons with pulse energy improve by one order of magnitude comparing to the conventional sub-nano-Joule solitons^5,6^.

In order to gain robust control of soliton dynamics and better power-scalable capability, studies on dissipative solitons in the cavity with large normal dispersion have attracted intense interests in fiber laser community recently^7–10^. In contrast to the conventional solitons or dispersion-managed solitons whose underlying dynamics solely roots in the phase modulation, dissipative solitons essentially experience both phase and amplitude modulations, by including large monotonic nonlinear chirping with spectral filtering as well as the nonlinear and dispersive phase accumulations^11,12^. Dissipative solitons are proven to be significantly tolerant of strong intra-cavity nonlinearity and excellent candidates for developing power-scalable ultrafast fiber lasers^13^. However, the presence of largely accumulated nonlinear chirping in dissipative solitons often leads to heavily chirped outputs, which need to be de-chirped out of the laser system for obtaining transform limited pulses.

A recent publication by Mao et al.^14^ has now introduced a novel birefringence-managed soliton (BMS) mechanism in normal dispersion cavity. By utilizing phase matching induced bandwidth control in polarization-maintained fiber segment, this recent work reports on the generation of self-retaining transform-limited solitary pulses without external-cavity de-chirping. Different from the use of polarization-maintained fiber as spectral filter in highly chirped dissipative solitons^15^, this work directly demonstrated that the birefringence managed phase matching could lead to effective tailoring of two orthogonally polarized components both in spectral and temporal domains. As shown in Fig. 1, the interplay between nonlinearity, dispersion and amplitude modulations can be confined within the bandwidth determined by the birefringence-involved phase matching and eventually results in steady-state soliton solution. The soliton formation dynamics is experimentally evidenced by dispersive Fourier transform measurements in the published work. The spectral profiles, evolutions and frequency resolved optical gating traces also explicitly show the distinct characteristics of BMS in comparing to conventional dissipative solitons with parabolic spectra. Intriguingly, based on length selection of polarization-maintained fiber segment, the proposed laser system can readily reach alternate solitary wave states between BMS and dissipative soliton. Yet there will be remaining technical challenge of improving the working bandwidth of BMS.

**Fig. 1:**
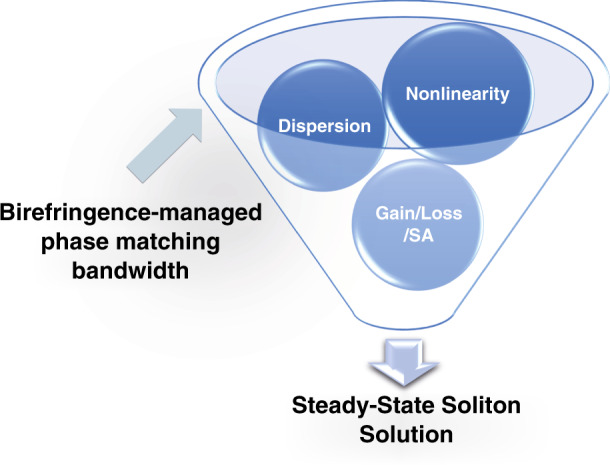
Principle of BMS Illustration of the key elements involved in birefringence-managed soliton formation

The technical findings reported in this work enrich the fundamental study of soliton dynamics and will find promising applications in different research areas related with soliton generation and dissemination. For example, BMS framework will enable future exploitations for generating power-scalable transform-limited ultrafast pluses in fiber laser cavity with large normal dispersions. The passive transmission of BMS will also be a promising solution for optical fiber communications.
